# Predictors of Treatment Effectiveness for Youth with ASD and Comorbid Anxiety Disorders: It all Depends on the Family?

**DOI:** 10.1007/s10803-016-2956-5

**Published:** 2016-12-21

**Authors:** F. J. A. van Steensel, V. M. Zegers, S. M. Bögels

**Affiliations:** Child Development and Education, Research Priority Area YIELD, University of Amsterdam, Nieuwe Achtergracht 127, 1018 WS, Amsterdam, The Netherlands

**Keywords:** Autism spectrum disorder, CBT, Anxiety, Treatment effectiveness, Children

## Abstract

The study aimed to explore predictors of treatment effectiveness in a sample of 79 children with ASD who received cognitive behavioral therapy (CBT) for their anxiety disorders. Severity of anxiety disorders and anxiety symptoms were used to measure treatment effectiveness and was assessed pre-treatment, post-treatment, 3 months-, 1 and 2 years after CBT. Child characteristics and maternal anxiety did not predict treatment effect. Children with anxious fathers and children in ‘un-involved’ families had less anxiety symptoms at pre-treatment and displayed a less steep decline. Children from ‘authoritarian’ families showed higher pre-treatment anxiety levels but responded quite well to treatment. Findings stress the importance of parent (father) and family factors in the treatment of anxiety disorders in youth with ASD.

## Introduction

Prevalence of anxiety disorders in children with autism spectrum disorders (ASD) is high (van Steensel et al. 2011; White et al. 2009) and cognitive behavior therapy (CBT) is an effective treatment for anxiety disorders also for children with ASD (van Steensel and Bögels 2015; see meta-analysis of Sukhodolsky et al. 2013). However, not all children benefit equally from the intervention. More insight in predictors for treatment efficacy for this specific group of children may enhance treatment and optimize outcomes. Therefore, this study explored possible predictors of treatment efficacy for anxiety disorders in children with ASD.

CBT is considered an efficacious treatment for anxiety disorders in children without ASD (Bodden et al. 2008; Cartwright-Hatton et al. 2004; Ishikawa et al. 2007). However, approximately one-third of the treated children still meet criteria for an anxiety disorder after treatment (Cartwright-Hatton et al. 2004; Seligman and Ollendick 2011). The reason for this differential treatment response is not fully understood. Several studies have reported various (psychological) factors of the child and parent as predictors of CBT effectiveness in children without ASD. Research has examined the following child factors as possible predictors for treatment efficacy: age, internalizing psychopathology, pre-treatment comorbidity, depression and trait anxiety (Berman et al. 2000; Southam-Gerow et al. 2001). Parental psychological factors such as parental anxiety, depression, hostility, and paranoia have also been shown to predict treatment outcomes (Berman et al. 2000; Bodden et al. 2008; Creswell et al. 2008; Southam-Gerow et al. 2001). Lastly, a few family factors such as family dysfunction have been associated with poorer treatment outcomes (Crawford and Manassis 2001).

The identification of treatment predictors in child anxiety (without ASD), however, are not undisputed and research reports inconsistent findings. For example, Southam-Gerow et al. (2001) reported that older-child age was associated with less favourable treatment response, while in other studies age was not found to be a predictor for treatment efficacy (Berman et al. 2000; Kendall et al. 1997; Treadwell et al. 1995). In addition, Berman et al. (2000) found an association between treatment outcome and comorbidity, however, pre-treatment comorbidity was not found to be related to treatment outcome in several other studies (Kendall et al. 1997, 2001; Ollendick et al. 2008). The same inconsistent findings have been found for parental factors. For example, Berman et al. (2000) reported that parental anxiety was not found to predict child treatment outcomes, while other studies found that parental anxiety has been correlated with poorer treatment outcomes (Bodden et al. 2008; Creswell et al. 2008).

To the best of the author’s knowledge, there are only two studies to date that have examined possible predictors of treatment efficacy for children with ASD and comorbid anxiety disorders (Conner et al. 2013; Storch et al. 2015). The study of Conner et al. (2013) examined the relationship between parental anxiety and treatment response in adolescents with ASD. The results showed that children of more anxious parents responded equally well to treatment, however, parents of treatment responders did experience a decrease in their own anxiety while parents of treatment non-responders did not. The study of Storch et al. (2015) showed that (1) more family accommodation (defined as strategies/behaviours that family members use to avoid the anxious child to become anxious, distressed or to have outbursts) was related to more anxiety symptom severity, (2) family accommodation was decreased after CBT treatment, and (3) family accommodation was lower in treatment responders compared to non-responders. Noteworthy is also the study of Maddox et al. (2016) which did not evaluate treatment effectiveness based on anxiety but on social functioning. It was found that loneliness was not a significant predictor of change, but more social anxiety was related to social impairment as well treatment change. That is, individuals with more social anxiety—as compared to individuals with less social anxiety—had (1) poorer social functioning at pre-treatment, (2) demonstrated more improvement during treatment, but also (3) tended to deteriorate between treatment endpoint and the 3-month follow-up (Maddox et al. 2016).

More insight in the factors that play a role in treatment efficacy is needed and important for theory development and clinical practice. The process of isolating these variables and their relationship to treatment outcomes will enable professionals to match individual children to specific treatment programs (Sherer and Schreibman 2005) and thereby improve overall treatment efficacy. This study used the same ASD sample as described in van Steensel and Bögels (2015) for which standard CBT was found to be effective for anxiety problems up to 2 years after treatment, and was not found to be very differently effective compared to a non-ASD sample. However, individual differences in treatment responding were also found. That is, in the current study, about 60% of the children with ASD were free from their primary anxiety disorder and about 40% were free from all anxiety disorders at the 2 years follow-up, which implies that some children benefited more than others from the treatment. Therefore, the aim of the current study was to examine which pre-treatment characteristics are related to treatment effectiveness. The research questions of the study were: (1) Are child characteristics (i.e., gender, age, and child psychopathology) related to treatment outcome? (2) Does parental anxiety (i.e., clinical anxiety levels of mothers and fathers) predict treatment outcome? (3) Is family type (i.e., un-involved families, authoritarian families, indulgent families, and authoritative families) predictive for treatment outcome?

## Method

### Participants

The total sample consisted of 79 children with ASD and comorbid anxiety disorders (58 boys, *M*_age_ = 11.76, *SD* = 2.68; range = 7–18 years), 78 mothers and 57 fathers. Of the 79 children with ASD, 14 were classified with a DSM-IV-TR diagnosis of autistic disorder, 16 with Asperger’s disorder and 50 with PDD-NOS (of note, the DSM-IV-TR was the most current DSM at the time of the study). The Autism Diagnostic Interview-Revised (ADI-R; Lord et al. 1994) was administered to the parents of 60 children (76% of the total sample; of note, the instrument was added in a later phase of the research due to time issues regarding the translation of the instrument in Dutch and training). Percentages of children meeting ADI-R cutoff for the social, communication and repetitive domain were 97, 88 and 70% respectively. The ADIS-C/P (Anxiety Disorder Interview Schedule-Child/Parent version; Silverman and Albano 1996) was used to assess anxiety disorders. All children were found to meet ADIS-C/P criteria for at least one anxiety disorder, however, most children had multiple anxiety disorders (*M* = 5.51, *SD* = 3.24). Primary anxiety disorders (i.e., the child’s most impairing anxiety disorder) in the ASD group consisted of: specific phobia (38%), social anxiety disorder (30%), generalized anxiety disorder (16%), separation anxiety disorder (13%), agoraphobia (1%), and panic disorder (1%). Comorbid anxiety disorders (next to the primary anxiety disorder) were also frequently present, the most common ones being specific phobia (47%), generalized anxiety disorder (43%), and social anxiety disorder (32%). Mothers had a mean age of 42.71 years (*SD* = 4.66) and respectively 38, 35 and 27% had a low, middle, and high education level. The mean age of fathers was 44.75 years (*SD* = 4.93) and respectively 21, 40 and 39% had a low, middle and high educational level. For more information about the sample, see van Steensel and Bögels (2015).

### Procedure

Medical and ethical approval was given by the medical-ethical committee of the University of Maastricht/Academic hospital of Maastricht, and by the Ethics Review Board of the faculty of social and behavioral science of the University of Amsterdam. Written informed consent was acquired from all parents and from children aged 12 years or above. All children had been referred to secondary mental health care clinics of which the multi-disciplinary teams established the clinical DSM-IV-TR diagnoses. The CBT that was given is a combined version of the family and child CBT (Bögels and Siqueland 2006; Bodden et al. 2008) and is described in van Steensel and Bögels (2015). In short, the CBT consists of 15 sessions of 60 min and contains components such as psycho-education, relaxation and coping techniques, cognitive restructuring, exposures, and behavioral experiments. All 79 families completed pre-assessment (before CBT started), 76 were assessed at post-assessment (directly after CBT ended), 66 at follow up-1 (3 months after CBT), 63 at follow up-2 (1 year after CBT) and 58 at follow up-3 (2 years after CBT). Assessment took place either at the mental health care center or at the family’s home. All children were verbally able to complete the measurements. Assessments were conducted by psychologists/diagnosticians who were independent from the staff that diagnosed or treated the children. Inclusion criteria for the ASD group were: (1) having an DSM-IV-TR diagnosis of ASD (autistic disorder, Asperger, or PDD-NOS) as established by the multi-disciplinary team of the mental health care centers, (2) having at least one anxiety disorder (confirmed by the ADIS-C/P), and (3) at least one parent willing to participate. Exclusion criteria were: (1) IQ < 70, (2) un-treated psychotic disorder, (3) acute suicidal risk, and (4) current physical or sexual abuse. For more information, see van Steensel and Bögels (2015).

### Instruments used for Treatment Effectiveness

The ADIS-C/P (Silverman and Albano 1996) is a DSM-IV based semi-structured interview with good psychometric properties (Silverman et al. 2001) and has been used in ASD samples to assess anxiety disorders (e.g., Reaven et al. 2012; Wood et al. 2009). The ADIS-C/P follows a DSM-IV symptom check, followed by an impairment rating (i.e., a severity score from 0 to 8). A severity score of 4 or higher warrants a diagnosis. A total anxiety disorder severity score can be calculated by summing the severity scores of all anxiety disorders and has been used in previous research to address treatment effectiveness (e.g., Hudson et al. 2009; Kendall et al. 2008; Simon et al. 2011; van Steensel and Bögels 2015).

The SCARED-71 (Bodden et al. 2009) measures anxiety symptoms, has 71 items and a 3-point rating scale (0 = almost never, 1 = sometimes, 2 = often). An example item: ‘I am afraid of heights’. It has a self-report version and a parent version, and both versions were administered in the current study. Next to a total score, subscale scores can be derived for separation anxiety disorder, social anxiety disorder, specific phobia, generalized anxiety disorder, panic disorder, obsessive–compulsive disorder, and post-traumatic stress disorder. The psychometric properties of the SCARED-71 have been investigated in a typically developing sample with and without anxiety disorders (Bodden et al. 2009) and an ASD sample (van Steensel et al. 2013), and are found to be good. Cronbach’s alpha’s for the SCARED-71 in the current study ranged between 0.91 and 0.95 across the different assessments.

### Instruments used for Pre-Treatment Predictors

#### Child Characteristics

Child gender and age was measured with a demographic questionnaire. Child psychopathology was measured with the Child Behavior Check List (CBCL; Achenbach 1991), which consists of 112 behavioral items rated by parents on a 3-point scale (0 = not true, 1 = somewhat or sometimes true, 2 = very true or often true). Items can be summed into a total score, two broadband scales (internalizing and externalizing problems), and eight subscales (withdrawn/depression, somatic complaints, anxiety/depression, social problems, attention problems, thought problems, rule breaking behavior and aggressive behavior). In this study, the two broadband scales measured at the pre-assessment were used as a proxy of the child’s internalizing and externalizing psychopathology. The CBCL has good psychometric properties (Achenbach 1991) and Cronbach’s alpha in the current study was 0.88 for internalizing as well as externalizing problems.

#### Parental Anxiety

The SCARED-Adult version (SCARED-A; Bögels and van Melick 2004) was used to measure self-reported anxiety symptoms of fathers and mothers at pre-assessment. The SCARED-A has the same number of items and uses the same 3-point rating scale as the SCARED-71. A total score as well as subscales scores corresponding with the SCARED-71 can be calculated. Psychometric properties of the SCARED-A are found to be good and cutoffs indicative for clinical anxiety were established by van Steensel and Bögels (2014). These cutoffs (a total score of 30 or higher for mothers, and a total score of 20 or higher for fathers) were used in the current study to indicate clinical anxiety levels. Cronbach’s alpha in the current study for mothers was 0.93, and for fathers 0.94.

#### Family Type

Family functioning was assessed at pre-treatment with the Family Functioning Scale (FFS; Bloom 1985) which is a factor-analytic version of four family questionnaires: the Family Environment Scale, the Family Concept Q Sort, the Family Adaptability and Cohesion Evaluation Scales, and the Family Assessment Measure. The FFS is a questionnaire that was completed by parents and children, and contains 60 items rated on a 4-point scale (1 = very untrue; 2 = fairly untrue; 3 = fairly true; 4 = very true). Two dimensions can be derived: family relationship and system maintenance. A higher score on the dimension family relationship indicates a more cohesive, expressive, outgoing and supportive family. An example item is: ‘family members really helped and supported one another’. A higher score on the dimension system maintenance—also referred to as the dimension of ‘family control’ (Jongerden and Bögels 2015)—indicates a less organized, more hierarchical (and authoritarian) family with a higher external locus of control and more enmeshment. An example item is: ‘there was strict punishment for breaking rules in our family’. The dimensions of the FFS are found to have satisfactory psychometric properties (Bloom 1985). Cronbach’s alpha in the current study for family relationship was 0.95 and 0.83 for system maintenance/family control.

We have made four categories (family types) based on the interaction between the two FFS dimensions. We used this approach because Maccoby and Martin (1983) in Foxcroft and Lowe (1995) propose that certain family types may be more functional than others. They suggest for example that a family with a combination of high control and high support (i.e., authoritative families) is the most optimal family environment for children and adolescents, while the families with the combination of low control and low support (i.e., un-involved families) would possibly provide the most dysfunctional family environment. The four family types were made by dichotomizing the two dimensions of the FFS (family relationship *M* = 90.22, *SD* = 9.84; and system maintenance/family control *M* = 60.82, *SD* = 6.17, the correlation (*r*) between the two dimensions was −0.54, *p* < 0.001); by splitting the sample scores in half. Based on the categorization of the parenting patterns by Maccoby and Martin (1983) in Foxcroft and Lowe (1995) and following the categorization of Foxcroft and Lowe (1995), families were divided in one of four family types: (a) ‘un-involved families’ (n = 16): families scoring relatively low in both dimensions (being less supportive, expressive and cohesive in combination with being relatively undemanding and exerting less control), (b) ‘authoritarian families’ (n = 23): families scoring relatively low on family relationship (being less supportive, expressive and cohesive) but relatively high on system maintenance/family control (exerting high control), (c) ‘indulgent families’ (n = 20): families scoring relatively high on the relationship dimension (being more supportive, expressive, and cohesive) and relatively low on system maintenance/family control (being more permissive, exerting less control), and (d) ‘authoritative families’ (n = 16): families scoring relatively high on both dimensions (being both more supportive, expressive and cohesive in combination with being more demanding and exerting higher control).

### Analyses

Multi-level analyses were used to examine which variables were important for predicting treatment effectiveness. Multi-level analyses can be used when data is nested; in this study assessments (pre, post and follow-up’s) were nested within respondents (children, mothers, and fathers), and respondents were nested within families. Multi-level analyses account for these dependencies among assessments and respondents. An additional benefit of multilevel methods is that missing data is not an obstacle for performing the analyses. All available data is used, also data from families in which only one parent participated or families in which one or more assessments were missing (e.g., uses the pre-assessment data when post- or follow-up data is missing, or uses child and mother report when father report is missing). All variables were transformed to standardized scores. In this way the parameter estimates for continuous variables can be interpreted as *r* (0.1 = small, 0.3 = medium, 0.5 = large; Cohen 1992) and parameter estimates for dichotomous variables can be interpreted as Cohen’s *d* (0.3 = small, 0.5 = medium, 0.8 = large; Cohen 1992).

Two effectiveness measures were used as dependent variables in the two-level hierarchical models: the total anxiety severity score as measured by the ADIS-C/P, and the total anxiety symptom score as measure by the SCARED-71. Post-assessment, follow-up 1, follow-up 2 and follow-up 3 were entered as predictors to evaluate the change of anxiety severity over time (i.e., treatment effectiveness). Each predictors of interest [child characteristics (gender, age and child psychopathology), parental anxiety, and family type (one family type as contrasted against the other family types)] were analyzed in separate models to examine (1) their relation with anxiety severity in general (i.e., main effect), and (2) their influence on treatment effectiveness (i.e., interaction effect between the predictor and the different assessments).

## Results

### Treatment Effectiveness

In all models, significant effects of assessments (post-assessments and follow-ups) were found, indicating that the total severity of anxiety disorders (ADIS) and anxiety symptoms (SCARED) were decreased at the different assessments (after having followed CBT). Parameter estimates (interpretable as Cohen’s *d*) of the assessments in the different models ranged between −0.81 and −2.01 for the total anxiety disorder severity score (ADIS), and between −0.62 and −1.08 for the total symptom score (SCARED), indicating large treatment effects. For a detailed analysis of the overall effect of CBT, see van Steensel and Bögels (2015).

### Predictors

#### Child Characteristics

No significant effects were found for child gender or age (Table 1), indicating that gender or age is not related to anxiety severity or treatment effectiveness. A significant relation between anxiety disorder severity (ADIS) and externalizing problems was found as well as a significant relation between anxiety symptoms (SCARED) and internalizing problems (Table 1). These findings indicate that more externalizing problems measured at pre-treatment are associated with more severe anxiety disorders, and that more internalizing problems measured at pre-treatment are related to higher anxiety symptoms. Note however that the effect size (parameter estimates interpretable as *r*) was small for both effects and that no interaction effects were found to be significant, indicating that child psychopathology measured at pre-assessment was not related to treatment effectiveness.

**Table 1:** Child characteristics (gender, age, internalizing and externalizing behaviours) as predictors of anxiety treatment effectiveness of CBT for children with autism spectrum disorders

	Anxiety disorders (ADIS)	Anxiety symptoms (SCARED)
	Parameter estimate (SE)^a^	Parameter estimate (SE)^a^
Gender (0 = boy; 1 = girl)	−0.23 (0.25)	0.15 (0.14)
Gender X post	0.13 (0.19)	−0.07 (0.11)
Gender X follow-up 1	0.12 (0.21)	0.20 (0.15)
Gender X follow-up 2	0.21 (0.20)	0.13 (0.16)
Gender X follow-up 3	0.24 (0.24)	0.06 (0.20)
Age	0.14 (0.11)	−0.04 (0.06)
Age X post	−0.07 (0.08)	−0.00 (0.05)
Age X follow-up 1	−0.10 (0.09)	−0.04 (0.07)
Age X follow-up 2	−0.11 (0.09)	0.00 (0.07)
Age X follow-up 3	−0.20 (0.10)	0.15 (0.09)^#^
Internalizing problems	0.01 (0.01)	0.04 (0.01)**
Internalizing problems X post	0.01 (0.01)	−0.01 (0.01)
Internalizing problems X follow-up 1	0.01 (0.01)	−0.01 (0.01)
Internalizing problems X follow-up 2	−0.01 (0.01)	−0.01 (0.01)
Internalizing problems X follow-up 3	0.00 (0.01)	−0.01 (0.01)
Externalizing problems	0.03 (0.01)*	0.00 (0.01)
Externalizing problems X post	−0.01 (0.01)	0.00 (0.01)
Externalizing problems X follow-up 1	−0.01 (0.01)	0.00 (0.01)
Externalizing problems X follow-up 2	−0.02 (0.01)^#^	0.01 (0.01)
Externalizing problems X follow-up 3	−0.02 (0.01)	0.00 (0.01)

*p < 0.05; **p < 0.01; ^#^ *p* < 0.10; ^a^Parameter estimates can be interpreted as Cohen’s *d* (dichotomous predictors) or *r* (continuous predictors); Post = post-assessment (directly after CBT); Follow-up 1 = three months after CBT; Follow-up 2 = one year after CBT; Follow-up 3 = two years after CBT

#### Parental Anxiety

No significant effect for maternal anxiety was found, however, significant effects were found for paternal anxiety, see Table 2. It was found that children of anxious fathers had less severe anxiety disorders (ADIS) than children of non-anxious fathers. In addition, the interaction between assessment and paternal anxiety yielded significance for all follow-ups indicating that the severity of the anxiety disorders was decreased less for the children who had anxious fathers compared to the children who had non-anxious fathers. Note however that children of anxious fathers had less severe anxiety disorders overall and that the endpoint severity of both groups is similar. Considering anxiety symptoms (SCARED), it was found that the anxiety symptoms of children of anxious fathers were less decreased at follow-up 1 (3 months after treatment) compared to the anxiety symptoms of children of non-anxious fathers. However, for the other assessments, such an effect was not found.

**Table 2:** Parental anxiety as predictor of anxiety treatment effectiveness of CBT for children with autism spectrum disorders

	Anxiety disorders severity (ADIS)	Anxiety symptoms (SCARED)
	Parameter estimate (SE)^a^	Parameter estimate (SE)^a^
Mother anxiety (0 = not clinical; 1 = clinical)	0.06 (0.26)	0.16 (0.16)
Mother anxiety X post	−0.01 (0.22)	0.05 (0.12)
Mother anxiety X follow-up 1	0.32 (0.22)	0.16 (0.16)
Mother anxiety X follow-up 2	−0.12 (0.20)	0.30 (0.16)
Mother anxiety X follow-up 3	−0.01 (0.24)	−0.10 (0.22)
Father anxiety (0 = not clinical; 1 = clinical)	−0.82 (0.26)*	0.01 (0.16)
Father anxiety X post	0.37 (0.22)	−0.10 (0.12)
Father anxiety X follow-up 1	0.66 (0.21)*	0.29* (0.16)
Father anxiety X follow-up 2	0.53 (0.20)*	0.03 (0.16)
Father anxiety X follow-up 3	0.98 (0.24)**	0.11 (0.22)

*p < 0.05; **p < 0.01; Parental anxiety was found to be decreased after child CBT, however, the pre-post difference score of parental anxiety was not associated with CBT effectiveness; ^a^Parameter estimates can be interpreted as Cohen’s *d* (dichotomous predictors) or *r* (continuous predictors); Post = post-assessment (directly after CBT); Follow-up 1 = three months after CBT; Follow-up 2 = one year after CBT; Follow-up 3 = two years after CBT

#### Family Type

None of the family types were related to treatment effectiveness based on anxiety disorder severity (ADIS), however, several significant effects were found for anxiety symptoms (SCARED), see Table 3. Children of ‘un-involved families’ tended to have less anxiety symptoms compared to children of other families (borderline significant effect), however significant interaction effects between this family type and post-assessment, follow-up 1, and follow-up 2 (and a borderline significant effect for follow-up 3) were also found, indicating that anxiety symptoms of children in ‘un-involved families’ decreased less over time compared to children in other families. Children from ‘authoritarian families’—as compared to children from other families—had higher levels of anxiety symptoms overall, however, the significant interaction effects indicate that the anxiety symptoms of children from ‘authoritarian families’ were found to be decreased more at post-assessment and follow-up 1. One interaction effect was found significant between ‘indulgent families’ and follow-up 2, indicating that the anxiety symptoms of children from these families were more decreased at this assessment than the anxiety symptoms of children from other families. No significant effects were found for ‘authoritative families’.

**Table 3:** Family type as predictor of anxiety treatment effectiveness of CBT for children with autism spectrum disorders

	Anxiety disorders severity (ADIS)	Anxiety symptoms (SCARED)
	Parameter estimate (SE)^a^	Parameter estimate (SE)^a^
‘Un-involved family’ (0 = no; 1 = yes)	0.15 (0.28)	−0.31 (0.16)^#^
Un-involved family X post	−0.26 (0.21)	0.34 (0.12)*
Un-involved family X follow-up 1	0.03 (0.24)	0.58 (0.15)**
Un-involved family X follow-up 2	−0.10 (0.22)	0.60 (0.17)*
Un-involved family X follow-up 3	−0.21 (0.27)	0.40 (0.22)^#^
‘Authoritarian family’ (0 = no; 1 = yes)	0.24 (0.24)	0.46 (0.14)*
Authoritarian family X post	0.07 (0.19)	−0.49 (0.09)**
Authoritarian family X follow-up 1	−0.11 (0.21)	−0.37 (0.15)*
Authoritarian family X follow-up 2	−0.13 (0.20)	−0.27 (0.15)^#^
Authoritarian family X follow-up 3	−0.14 (0.23)	0.02 (0.19)
‘Indulgent family’ (0 = no; 1 = yes)	−0.03 (0.25)	−0.17 (0.15)
Indulgent family X post	0.01 (0.20)	0.07 (0.12)
Indulgent family X follow-up 1	−0.06 (0.22)	−0.15 (0.16)
Indulgent family X follow-up 2	0.09 (0.20)	−0.34 (0.16)*
Indulgent family X follow-up 3	−0.09 (0.25)	−0.27 (0.20)
‘Authoritative family’ (0 = no; 1 = yes)	−0.41 (0.27)	0.09 (0.16)
Authoritative family X post	0.15 (0.21)	0.06 (0.13)
Authoritative family X follow-up 1	0.16 (0.23)	−0.15 (0.18)
Authoritative family X follow-up 2	0.16 (0.22)	0.09 (0.18)
Authoritative family X follow-up 3	0.47 (0.26)	−0.10 (0.23)

^#^ *p* < 0.10; *p < 0.05; **p < 0.01; ^#^ *p* < 0.10; ^a^Parameter estimates can be interpreted as Cohen’s *d* (dichotomous predictors) or *r* (continuous predictors); Post = post-assessment (directly after CBT); Follow-up 1 = three months after CBT; Follow-up 2 = one year after CBT; Follow-up 3 = two years after CBT

## Discussion

This exploratory study was the first to examine multiple predictors for anxiety disorder treatment effectiveness in children with ASD. Effects of several pre-treatment characteristics on the severity of children’s anxiety disorder and the intensity of the anxiety symptoms were investigated after treatment and 3 months, 1 and 2 years after completing treatment. Interesting, child characteristics (gender, age and children’s internalizing and externalizing problems) were not found to have an influence on treatment effectiveness, however, parental and family predictors did. Most consistent findings across assessments were found for paternal anxiety and family type (more specifically, children growing up in an ‘uninvolved’ or ‘authoritarian’ family). The findings related to each of these predictors will be discussed in more detail below.

Children with ASD and comorbid anxiety disorders who have anxious fathers were found to have less severe anxiety disorders than children without anxious fathers. This result seems somewhat contra intuitive, however, it is possible that families with anxious fathers reach out for treatment and professional help sooner than families without anxious fathers. Reasons for this could be that these families recognise the signs of an anxiety disorders faster, that they cannot cope with the stress and problems of having of an additional member with anxiety disorders in the family, or that the father feels he is less able to full-fill his role as a parent. In line, research demonstrated that parents’ anxiety level may influence their expectations and cognitions about their child’s emotional and behavioural reactions to anxiety provoking situations (Cobham et al. 1999), that parental locus of control and perceived control of child anxious behaviour is affected when a parent is anxious (Wheatcroft and Creswell 2007), and that parental beliefs (about their children’s anxious disposition) may be important predictors of parental behaviour and may impact the parenting strategies they use (Bögels and Brechman-Toussaint 2006).

Additionally, the analyses revealed that the anxiety symptom scores and severity of anxiety disorders of children with ASD who have non-anxious fathers decreased more compared to the anxiety severity scores of children with anxious fathers at follow-up measurements, which is in line with studies that have found parental anxiety to be a predictor of less positive treatment outcomes in clinically anxious youth without ASD (Bodden et al. 2008; Creswell et al. 2008). However, it is important to keep in mind that at the final follow-up assessment the anxiety levels of the two groups were very similar, and therefore it cannot be ruled out that the decrease for children with ASD and comorbid anxiety disorders who have non-anxious fathers is relatively strong because these children had more severe anxiety scores to begin with. Thus, having an anxious father might not necessarily be a predictor for negative treatment outcomes, but instead having an anxious father may make a family more likely to seek help with lower anxiety levels. Alternatively, or additionally, as we have only measured parental anxiety and not parental ASD symptoms, it is possible that the higher anxiety symptoms in fathers also reflect the presence of ASD symptoms in fathers. It is interesting to note, however, that no influence of maternal anxiety on anxiety severity or treatment outcomes was found.

The finding that paternal, but not maternal, anxiety predicts child anxiety treatment outcome is firstly consistent with theories about the important role of the father in the development and maintenance of anxiety disorders, and in overcoming childhood anxiety disorders, that is, by challenging the child to take risks and explore novel territory, and as such help them overcome anxiety and develop confidence (Bögels and Phares 2008; Bögels and Perotti 2011). An additional explanation of the finding that paternal, but not maternal anxiety predicts treatment outcome of anxiety in children with ASD concerns the function of fathers for children with autism. Fathers in general are found to suffer less from offspring diagnosed with ASD (see review of Karst and Vaughan Van Hecke 2012) perhaps because they expect less mutuality in contact. Related, fathers may be more capable in interacting with children with ASD, as fathers generally tend to do activities with their children, whereas mothers tend to socialise with their children (e.g., Bögels and Phares 2008; Möller et al. 2013) an area which is more difficult with children and adolescents with ASD. ASD has also been conceptualised as an extreme male brain (Baron-Cohen 2002), and so fathers may be more similar to and therefore more understanding of their ASD child. From here, it can be speculated that anxious fathers are less capable in fulfilling the paternal role that children with ASD need, for example to stimulate them to explore novel territory, as children with ASD tend to insist on sameness.

It was found that children with ASD and comorbid anxiety disorders who live in ‘un-involved families’ (families with a relatively low score on family relationship and system maintenance/family control)—as compared to other family types—tended to have less anxiety symptoms, but also the anxiety symptoms decreased less over time for these children. Un-involved families are characterized by being less supportive, cohesive and expressive (low on family relationship; Bloom 1985) and provide little control, are relatively undemanding and have a less hierarchical family structure (low on system maintenance/family control; Bloom 1985). It might be that because these family members are less cohesive, less involved with each other, and less likely to express their feelings, they recognize and report less anxiety symptoms in their children. In addition, being not so much involved with each other, being less supportive and expressive in combination with having relatively few rules and less hierarchy may be less beneficial for children with ASD overcoming their anxiety as for them a clear structure and explicit explanations of feelings, social expectations and emotions might be important to be able to understand and predict (social) situations more easily and to be able to function well. Additionally, having supportive family members might help children overcome their fears more easily and help them to prevent possible relapse, for various reasons, as supportive families may help more with the CBT homework, may help with generalisation of learned CBT skills, and may provide more of a sense of safety for the child, which may be a precondition to learn new skills.

Children with ASD and comorbid anxiety disorders from ‘authoritarian families’ were found to have more anxiety symptoms compared to children from other families. Authoritarian families are characterized by being more hierarchical and exerting more control (high on system maintenance/family control; Bloom 1985) in combination with being somewhat less supportive, cohesive and expressive (low on family relationship; Bloom 1985). Interesting, high control has been related to anxiety in children in non-ASD populations (Van der Bruggen et al. 2008), and the current study seems to confirm this finding for children with ASD. However, although children with ASD and comorbid anxiety disorders living in authoritarian families have higher levels of anxiety compared to the other families to begin with, they do respond well to treatment and show a rather fast decrease in anxiety symptoms. That is, at post-test and follow-up 1 the anxiety symptoms of children from authoritarian families were found to be decreased more—resulting in similar anxiety levels—compared to children from other families. It might be that the structure and control provided by authoritarian families works quite well for children with ASD and comorbid anxiety disorders when they have to learn new skills and face new challenges (i.e. overcoming fears) as these families may provide more clear rules, guidelines and structure, and/or may stick better to the rules, guidelines and structure of the CBT. In addition, having learnt how to cope with anxious feelings may have provided the children with ASD (and their parents) a more clear format about how to express, understand and communicate about their (anxious) feelings, which may have led to a rather quick decrease in anxiety symptoms.

Limitations of the study need to be considered. First, due to the relatively small sample size and the complexity of the models, it was not possible to enter multiple predictors at the same time and therefore multiple models were run with each predictor analysed separately. Therefore it was also not possible to examine which predictor might have more or less influence, or if predictors interact with each other. Second, the four family types that have been used in this study were based on the sample means of the two dimensions of the FFS because no cut-offs for these dimensions exist. Therefore, it is unclear to what extent the family types that were used in this study represent extremes or just mild variants of the family types. Third, other factors, such as child IQ or other parent psychopathology than anxiety, such as ASD symptoms, that were not accounted for in this study may be related to treatment effectiveness. Lastly, the group of children with ASD could not be compared to a control group of children who did not receive treatment. Therefore, it is unsure whether paternal anxiety and family type are impacting (long-term) treatment effects or if these factors are impacting the natural progression of anxiety over time. However, the results are still important as it leads to new hypotheses for future research and a better understanding of the relation between anxiety (treatment) and ASD.

This study was the first to assess multiple predictors for the effectiveness of anxiety treatment for youth with ASD. Strengths of the study include the clinical nature of the sample (children were referred to community health care centres not specifically specialized in ASD or anxiety disorders), the inclusion of family predictors, and the long term assessments. More insight in predictors of treatment efficacy can lead to treatments that are better suited for individual children and to optimize treatment effectiveness. Unfortunately, predictors that were found significant in the current study varied with respect to their consistency across assessments and across effectiveness measures (i.e., effectiveness measures based on anxiety disorder severity versus anxiety symptom severity), which makes it difficult to draw firm conclusions. The difference across effectiveness measures may have to do with the way effectiveness and the predictor is measured, and how much these measures are alike. That is, paternal anxiety (defined as clinically anxious fathers) and effectiveness related to anxiety disorder severity (ADIS) may be both more specific and disorder-like, while the underlying dimensions of the family types and the effectiveness related to anxiety symptoms (SCARED) may be somewhat broader. In addition, family functioning may be more related to symptoms and behaviors than to (specific) disorders. Despite the inconsistency, the results of this exploratory study generates new hypotheses for future research and identifies challenges for clinical practice. More specific, the study findings suggest that child characteristics may have less impact on treatment effectiveness for children with ASD and comorbid anxiety disorders, while it highlights the importance of parent and family factors.
